# Relapsing meningitis and limbic encephalitis in anti-AQP4-Ab-associated neuromyelitis optica spectrum disorder

**DOI:** 10.1177/13524585241261549

**Published:** 2024-07-30

**Authors:** Giovanni Novi, Elvira Sbragia, Luana Benedetti, Angelo Schenone, Antonio Uccelli, Roberta Magliozzi, Massimo Del Sette, Matilde Inglese, Alice Laroni

**Affiliations:** IRCCS Ospedale Policlinico San Martino, Genova, Italy; Department of Neuroscience, Rehabilitation, Ophthalmology, Genetics, Maternal and Child Health (DINOGMI) and Center of Excellence for Biomedical Research (CEBR), University of Genova, Genova, Italy; Department of Neurology, Ospedali Galliera, Genova, Italy; IRCCS Ospedale Policlinico San Martino, Genova, Italy; Department of Neuroscience, Rehabilitation, Ophthalmology, Genetics, Maternal and Child Health (DINOGMI), University of Genova, Genova, Italy; IRCCS Ospedale Policlinico San Martino, Genova, Italy; Department of Neuroscience, Rehabilitation, Ophthalmology, Genetics, Maternal and Child Health (DINOGMI), University of Genova, Genova, Italy; IRCCS Ospedale Policlinico San Martino, Genova, Italy; Section of Neurology, Department of Neuroscience, Biomedicine and Movement Sciences, University of Verona, Verona, Italy; IRCCS Ospedale Policlinico San Martino, Genova, Italy; Department of Neuroscience, Rehabilitation, Ophthalmology, Genetics, Maternal and Child Health (DINOGMI), University of Genova, Genova, Italy; IRCCS Ospedale Policlinico San Martino, Genova, Italy; Department of Neuroscience, Rehabilitation, Ophthalmology, Genetics, Maternal and Child Health (DINOGMI), University of Genova, Genova, Italy; IRCCS Ospedale Policlinico San Martino, Genova, Italy

**Keywords:** NMOSD, meningitis, limbic encephalitis, eculizumab

## Abstract

**Objectives:**

neuromyelitis optica spectrum disorder (NMOSD) is a rare autoimmune disease mainly affecting optic nerves and the spinal cord. Due to the potentially irreversible tissue damage, prevention of relapses is of utmost importance.

**Methods:**

We describe the atypical clinical course and pathology results of a patient with anti-aquaporin-4 antibody (anti-AQP4-Ab)-associated NMOSD who developed aseptic meningitis followed by limbic-encephalitis-like presentation with extensive brain lesions upon treatment with rituximab and tocilizumab.

**Results:**

The patient developed subacute cognitive decline with magnetic resonance imaging (MRI) evidence of extensive brain white matter lesions. In the hypothesis of an opportunistic brain infection, she underwent brain biopsy of the temporal pole. Pathology results revealed typical NMOSD findings with complement activation, supporting the hypothesis of an atypical presentation of anti-AQP-Ab-associated NMOSD. Accordingly, treatment with the complement-targeting drug eculizumab was started, leading to a dramatic clinical and MRI improvement.

**Discussion:**

aseptic meningitis and limbic encephalitis could represent a rare phenotype of anti-AQP4-Ab-associated NMOSD.

## Introduction

Anti-aquaporin-4 antibody (anti-AQP4-Ab)-associated neuromyelitis optica spectrum disorder (NMOSD) is a rare autoimmune disease affecting the central nervous system (CNS).^1^ Optic nerves and spinal cord are the most frequently involved structures, due to the extensive expression of AQP4;^1^ however, NMOSD-associated lesions might also develop, in some cases, in circumventricular structures and within brain white matter.^2^

## Methods

We report the case of a 40-year-old woman with Hashimoto’s thyroiditis and essential tremor that was diagnosed as having anti-AQP4-Ab-associated NMOSD in November 2017, after left-side optic neuritis and subsequent thoracic myelitis with detection, by live cell-based assay, of anti-AQP4-Ab. Since diagnosis, rituximab treatment was initiated (two 1-g infusions 15 days apart) and subsequent 375 mg/m^2^ infusions (August 2018 and June 2019) were tailored according to memory B cells repopulation (as described by Kim et al.^3^).

## Results

**Figure 1.**
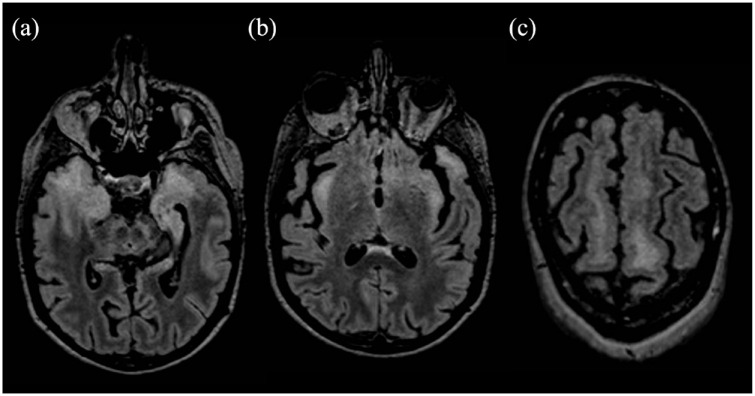
Fluid attenuated inversion recovery (FlAIR) sequences on the axial plane showing white matter hyperintensities involving the temporal lobe bilaterally (a), the insula (b), and frontal white matter (c).

Few weeks after last rituximab infusion, patient developed high-grade fever (39°C) and was then treated with antibiotics, without improvement. She presented with severe headache with signs of meningeal irritation. A lumbar puncture was performed, disclosing a marked T-cell pleocytosis (287 cells/mm^3^, normal range 0–5). After negative polymerase chain reaction and cultural examination, high-dose steroid (1 g/day methylprednisolone for 5 days), followed by intravenous human immunoglobulin (IVIG) (0.4 g/day for 5 days), was initiated, with rapid recovery. Due to the scarcity of venous access, which would have necessitated central venous catheter placement, use of IVIG was preferred over plasma exchange. A month later, meningeal irritation signs relapsed, and another course of high dose steroid was started, followed by oral prednisone treatment (25 mg/day). A complete workup was also performed, to exclude possible differential diagnosis: clinical examination, immunological tests, total-body CT scan, and fluorodeoxyglucose positron emission tomography scan did not show characteristics attributable to other autoimmune diseases (e.g. systemic lupus erythematosus, Behçet’s disease, sarcoidosis, and vasculitis). Anti-nuclear antibodies showed a low titer (1:160) with a speckled pattern, anti-double strand DNA antibodies, anti-extractable nuclear antigens antibodies, and anti-neutrophil cytoplasmic antibodies tested negative. In addition, angiotensin converting enzyme levels were within normal range. In addition, anti-thyroperoxidase and thyroglobulin antibodies in the patient’s serum and cerebrospinal fluid (CSF) tested negative. Furthermore, Mollaret’s meningitis was excluded on the basis of cytological examination of CSF (i.e. absence of Mollaret cells) and negative CSF polymerase chain reaction (PCR) test for Herpes Virus (especially Herpes-Simplex-2 virus and Varicella-Zoster virus). Subsequently, since relapsing meningitis was considered a possible atypical presentation of NMOSD relapse occurring during B cell-depletion treatment, tocilizumab treatment (8 mg/kg every 4 weeks) was initiated in February 2020. Low dose (5 mg/day) prednisone treatment was maintained due to two further relapse of meningeal signs irritation occurring upon discontinuation attempts. In November 2020, patient developed subtle cognitive changes with short-term memory loss and progressive daily life activities impairment. In addition, worsening of essential tremor was noted. Patient was unable to walk, perform simple tasks, and retain memories with clear short-term memory deficit. A brain magnetic resonance imaging (MRI) scan revealed extensive bilateral white matter lesions in the temporal, frontal, and insular areas, and in the cerebral peduncles (Figure 1). A lumbar puncture was performed to exclude infections, including progressive multifocal leukoencephalitis, and confirmed the presence of T cells (10 cells/mm^3^), without evidence of bacterial and/or viral infections. CSF PCR for *Listeria monocytogenes, Neisseria meningitidis, Haemophilus influenzae, Streptococcus pneumoniae*, Herpes Simplex virus (1 and 2), Varicella-Zoster virus, cytomegalovirus, Epstein–Barr virus, Adenovirus, Enterovirus, and John Cunningham virus tested negative twice. Culture test and Cryptococcus antigen test came back negative. After performing a full workup, including CSF and serum tests for anti-glial fibrillary acidic protein (GFAP) ab (negative), anti-myelin oligodendrocyte glycoprotein ab (negative), anti-neuronal membrane and intracellular ab (negative), a brain biopsy was carried out. Histology revealed presence of glial (GFAP +) cells with rare interspersed neuronal elements, showing mild and only focal increased astrocytic cellularity, without significant cytoarchitectural atypia, mitosis, vascular proliferation, or necrosis. In addition, “cuffs” composed of several small-sized lymphocytes, plasma cells and eosinophilic granulocytes were found around intraparenchymal microvascular blood structures.

**Figure 2.**
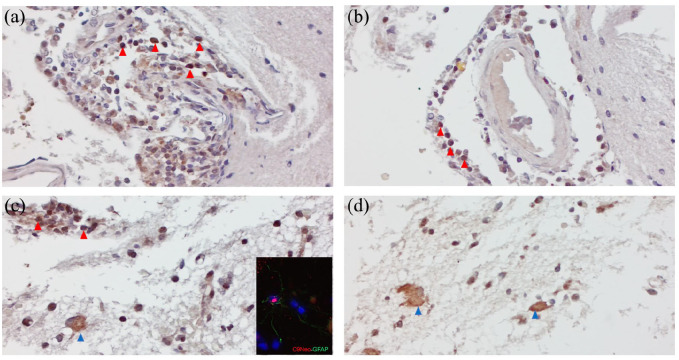
Neuropathological assessment of the expression of the complement activation marker C9Neo of brain biopsy: C9Neo immunostaining was prevalently detected on perivascular lympho-monocytic infiltrates (red arrows in a, b), but also on parenchymal cells morphologically resembling astrocytes (blue arrows in c, d), as confirmed by double immunofluorescence immunostaining with C9Neo and GFAP antibodies (inset in Figure 2(c)). Original magnifications: 200× (a, b), 400× (c, d, inset in c).

**Figure 3.**
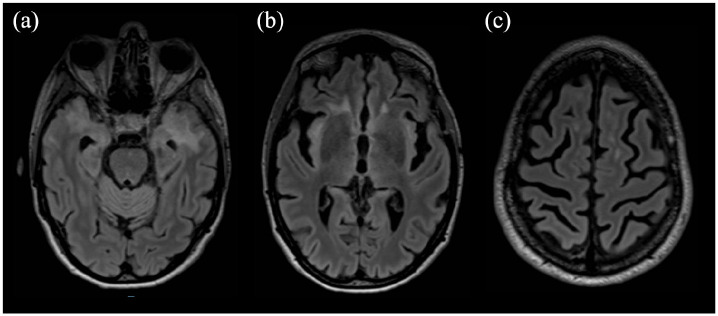
Fluid attenuated inversion recovery (FlAIR) sequences on the axial plane showing, after eculizumab initiation, a reduction of white matter hyperintensities involving the temporal lobe bilaterally (A), the insula (B), and frontal white matter (C).

In order to ascertain whether the white matter lesions were due to NMOSD activity disease, we performed further neuropathological assessment of formalin-fixed paraffin-embedded (FFPE) biopsy (7µ m) sections. We found that the expression of the complement activation marker C9Neo (Polyclonal Rabbit anti-human C9Neo antibody, Invitrogen) was prevalently on perivascular lympho-monocytic infiltrate (Figure 2(a) and (b)), but also on parenchymal cells morphologically resembling astrocytes (Figure 2(c) and (d)). Double immunofluorescence validated the presence of C9Neo + GFAP + astrocytes in the biopsy brain samples (inset in Figure 2(c)). Such results were consistent with typical NMOSD pathology. Following biopsy results, high dose steroid treatment (1 g methylprednisolone for 5 days) followed by intravenous immunoglobulins (0.4 g/day for 5 days) were administered, with partial cognitive improvement. Given that our analysis of the histological specimen demonstrated complement activation, and approval of anti-complement 5a (C5a) antibody for anti-AQP4-Ab-associated NMOSD by the European Medicine Agency (EMA), we started treatment with intravenous eculizumab (loading dose of 900 mg for 4 weeks followed by 1200 mg every 15 days), which led to immediate improvement. Ten months after treatment initiation, follow-up neurological examination and brain-MRI scans (Figure 3) showed clinical and radiological improvement, with reduction of brain lesions and recovery of cognitive performances (ability to carry out daily activities). In addition, steroid treatment was tapered and then discontinued without recurrence of disease. In consideration of ongoing steroid treatment at eculizumab initiation, and the potential risk for a failed vaccine response, prophylaxis for *N. meningitidis* infection, as recommended by EMA,^4^ with amoxicillin (500 mg twice daily) was initiated before eculizumab first administration. Patient was vaccinated against *N. meningitidis* (ACYW and B), 2 months after discontinuation of steroid therapy and amoxicillin treatment was withdrawn 2 weeks after vaccination schedule completion. At 24-month follow-up, no disease relapse occurred and further clinical improvement was observed, the patient is now independent in daily living.

## Discussion

Here, we report an atypical case of limbic encephalitis-like pattern with meningeal involvement of anti-AQP4-Ab-associated NMOSD, developed during treatment with CD20-depleting and subsequent anti-interleukin 6 receptor-blocking antibodies. Biopsy specimen analyses were consistent with NMOSD relapse diagnosis and supported the use of complement blocking antibody, leading to disease remission and partial symptoms regression. We advise neurologists and NMOSD specialists that rare instances of meningeal involvement and/or limbic encephalitis-like lesions might occur in NMOSD and could be refractory to conventional treatments, requiring complement blocking agents.
